# Antibiotic resistance patterns of environmental bacteria from sewage water in Vellore, India: isolation, virulence analysis, and characterization

**DOI:** 10.3389/fmicb.2025.1640369

**Published:** 2025-08-12

**Authors:** Surbhi Kumari Barnwal, Arabi Mohammed Saleh

**Affiliations:** ^1^School of Bio Sciences and Technology, Vellore Institute of Technology, Vellore, Tamil Nadu, India; ^2^VIT School of Agricultural Innovations and Advanced Learning, Vellore Institute of Technology, Vellore, Tamil Nadu, India

**Keywords:** antibiotic resistance, antibiotic resistance bacteria, amoxicillin, meropenem, vancomycin, sewage

## Abstract

Globally, the rise of antibiotic resistance is a pressing One Health concern, where environmental sources, particularly wastewater, play a critical role in the dissemination of resistant bacteria. The influx of pharmaceutical waste, likely to contain antibiotics, into the environment would lead to the chronic presence of antibiotics and development of resistance in environmental bacteria. This study aimed to investigate the prevalence and antibiotic resistance patterns of bacterial isolates obtained from sewage receiving hospital wastewater. Sewage samples were collected from four different locations in Vellore, Tamil Nadu, India. The samples were further analyzed using LC/MS for quantification of amoxicillin, meropenem, and vancomycin. The bacterial isolates were obtained by both direct and enrichment culture techniques. The isolates were phenotypically characterized by analyzing the colony morphology and through gram staining, and virulence tests (hemolysis assay, biofilm formation assay, and protease, amylase and lipase activity assays). Minimum inhibitory concentrations (MICs) against amoxicillin, meropenem, and vancomycin were determined using both antibiotic gradient strips and broth microdilution methods, following EUCAST guidelines. Molecular identification was performed using 16S rRNA gene sequencing. Although antibiotics were below the limit of quantification (BLQ) in the samples, significant resistance was observed among the isolates. A total of 10 bacterial strains, including *Stenotrophomonas, Sphingobium, Brucella, Agrobacterium, Ochrobactrum, Acinetobacter, Klebsiella*, and *Pandoraea* were identified. Most of the isolates exhibited multidrug resistance (MDR), with notable variability in MIC values (*p* < 0.05). *Pandoraea* sp. strain VITSA19 displayed the highest resistance to all the tested antibiotics (≥4,096 μg/mL for amoxicillin, ≥512 μg/mL for meropenem and ≥4,096 μg/mL for vancomycin). Two isolates, *Stenotrophomonas* sp. strain VITSA1 and *Stenotrophomonas pavanii* strain VITSA2, demonstrated hemolysin and protease production. These findings underscore sewage as a reservoir of MDR bacteria and highlight the environmental dimension of antibiotic resistance spread. From a One Health perspective, the study emphasizes the urgent need for integrated environmental antimicrobial resistance (AMR) surveillance and improved wastewater treatment practices to mitigate the risk of resistance transmission to human and ecological health.

## 1 Introduction

Antibiotic resistance has emerged as one of the most critical global health threats, undermining the efficacy of antibiotics and complicating the treatment of infectious diseases, and increasing the risk of infection (Ahmed et al., 2024). Antibiotic resistant bacteria (ARB) were directly responsible for 1.27 million deaths globally (Murray et al., 2022). The environmental dimension of antibiotic resistance, particularly the role of wastewater systems in harboring and disseminating resistant microorganisms, is increasingly recognized within the One Health framework. This highlights the interconnectedness of human, animal, and environmental health in the emergence and spread of antimicrobial resistance (AMR) (Manyi-Loh et al., 2018).

Wastewater systems serve as convergence points for antibiotics, antibiotic-resistant bacteria (ARB), and antibiotic resistance genes (ARGs), originating from human, agricultural, and industrial sources (Sambaza and Naicker, 2023). The uncontrolled use of antibiotics in healthcare, pharmaceutical industries, domestic, livestock farming, and agriculture contributes to their accumulation in wastewater, where they persist and interact with diverse microbial communities (Koch et al., 2021). The sub-inhibitory concentration of antibiotics has a non-negligible effect as it exert selective pressure on microbes that promotes the evolution and spread of resistance through mechanisms such as horizontal gene transfer (HGT) and mutation. HGT in particular, facilitates the rapid exchange of resistance genes via plasmids, integrons, and transposons that poses a major risk to public health (Laureti et al., 2013). The increasing threat of antibiotic resistance necessitates a comprehensive understanding of its prevalence and dissemination in wastewater and sludge environments.

Sewage often serves as reservoir and channel for antibiotics and its active antibiotic metabolites. Hence, it plays a pivotal role in antibiotic resistance dissemination. Inadequate treatment of such effluents before environmental discharge enables the release of ARB and ARGs into the natural water bodies, promoting their integration into environmental microbiomes (Fouz et al., 2020; Koch et al., 2021). Previous studies have shown a higher diversity and abundance of ARGs in hospitals effluents compared to other water sources, highlighting the urgent need for surveillance and management strategies targeting such point sources of resistance. For example, a study revealed that highest diversity of ARGs in effluent (1,520 unique genes) compared with river water and lake water (247 unique genes), highlighting the role of effluents as reservoirs of ARGs (Bondarczuk and Piotrowska-Seget, 2019). Wastewater surveillance offers a cost effective strategy to identify emerging resistance trends and evaluate the effectiveness of mitigation measures (Hassoun-Kheir et al., 2025). It also allows for the characterization of resistant bacterial species and their virulence potential, which are crucial for understanding their ecological and clinical implications.

The present study was conducted in order to validate the prevalent hypothesis that sewage receiving hospital wastewater harbors MDR bacterial populations, and that the presence of antibiotic residues contributes to the selective enrichment of these resistant strains, posing a potential threat to public health through environmental dissemination. Amoxicillin, a β-lactam antibiotic, is among the most widely consumed antibiotics globally (Browne et al., 2021; Akhavan et al., 2023). Meropenem, a carbapenem β-lactam, is frequently reserved for the treatment of MDR infections (Elshamy and Aboshanab, 2020). Vancomycin, a glycopeptide, is vital for treating resistant gram-positive infections, including methicillin-resistance *Staphylococcus aureus* (MRSA) (Gardete and Tomasz, 2014). Given their clinical relevance and contrasting resistance dynamics, these antibiotics provide a comprehensive perspective on resistance development in wastewater environments. In alignment with global concerns regarding the dissemination of antibiotic resistance through environmental reservoirs, this study investigated sewage samples from Vellore, an urban region in Tamil Nadu, India. The sampling site is situated in proximity to healthcare facilities, making it a plausible recipient of hospital effluents in addition to typical urban sewage inputs. This strategic location was selected to investigate the potential contribution of hospital-associated waste to the local burden of ARB. This study aims to isolate and identify bacterial species from sewage, determine their resistance profiles against amoxicillin, meropenem and vancomycin, evaluate the presence of virulence factors such as hemolysis and biofilm formation, assess the environmental concentrations of these antibiotics using LCMS and characterize the isolated bacteria by 16S rRNA sequencing. This integrated approach helps to elucidate the risk posed by environmental ARB in the context of public health and ecosystem safety.

## 2 Materials and methods

### 2.1 Materials

Amoxicillin trihydrate and meropenem trihydrate were purchased from Tokyo Chemical Industries (TCI), Hyderabad, India and vancomycin hydrochloride (Vanlid 500) was purchased from Cipla, Gujarat, India. All other chemicals used were of analytical grade. Potassium dihydroxy phosphate, dipotassium hydroxy phosphate, ammonium chloride, ferric chloride hexahydrate, EDTA, Mueller-Hinton agar, Brain Heart Infusion, nutrient agar (procured from Himedia, Mumbai, India), sodium sulfate, sodium molybdate, calcium chloride dihydroxy, zinc chloride, magnesium chloride dihydroxy, dextrose, DCM, sucrose (procured from SRL, Mumbai, India), Tween 80 (SDFCL, Mumbai, India), and methanol (HPLC and spectroscopy grade, FINAR, Chennai, India) were used in this study. All work was conducted under BSL-2 laboratory conditions. Table 1 shows the chemical formula, structure, and molecular weight of the antibiotics used in the study.

**Table 1:** Chemical formulas, structures, and molecular weights of the studied antibiotics.

**Antibiotics**	**Chemical formula**	**Structure**	**Molecular weight (g mol^−1^)**
Amoxicillin trihydrate	C_16_H_19_N_3_O_5_S	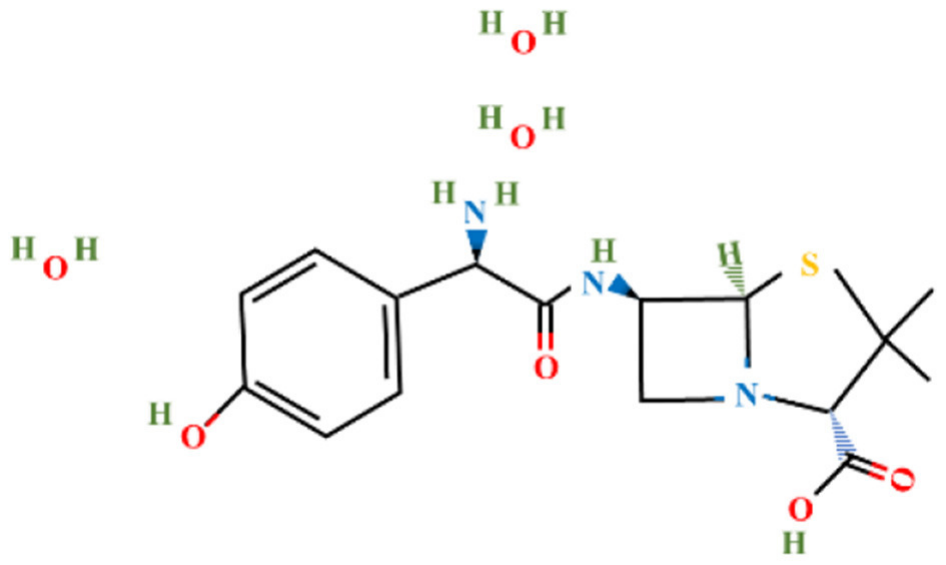	365.4
Meropenem trihydrate	C_17_H_31_N_3_O_8_S	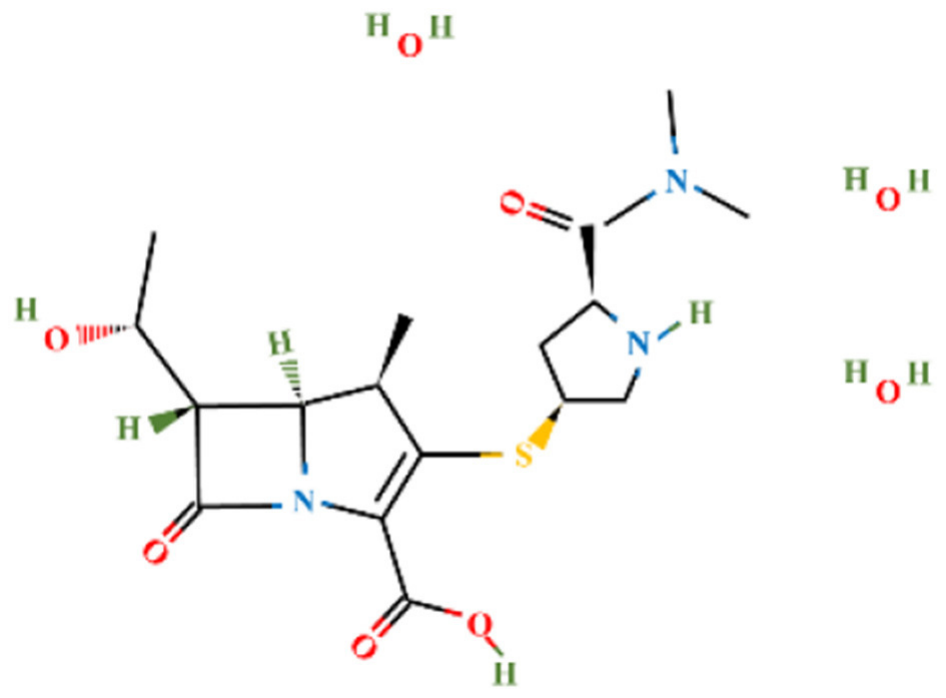	437.5
Vancomycin hydrochloride	C_66_H_76_C_l3_N_9_O_24_	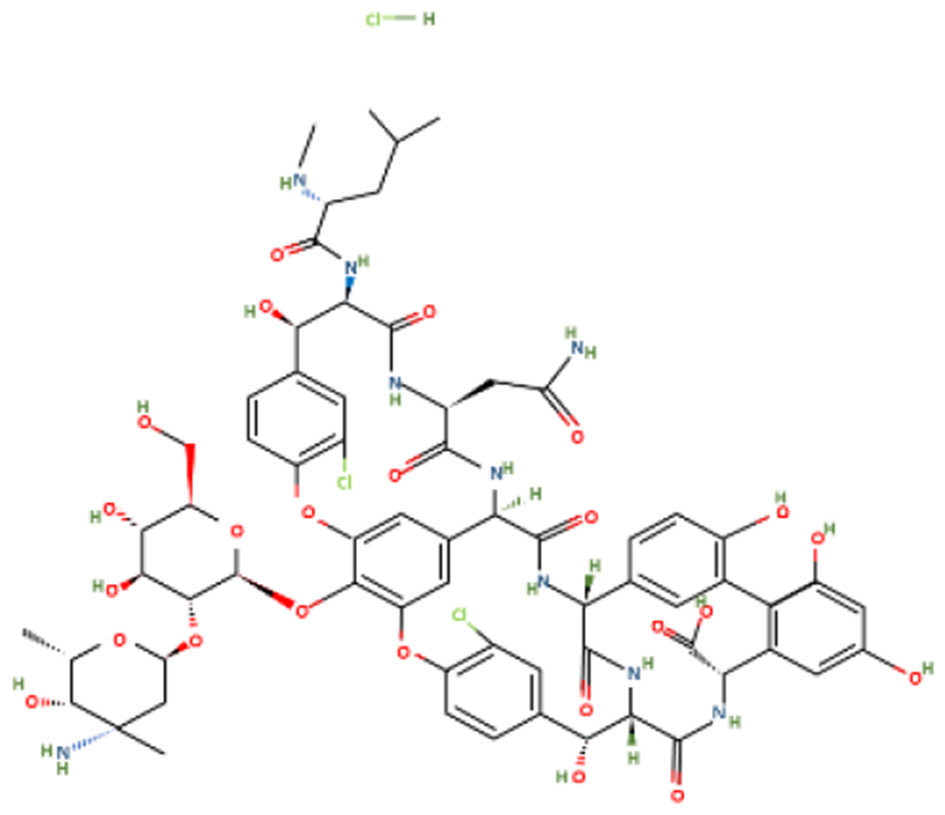	1,485.7

### 2.2 Sample collection

Sewage samples were collected from nearby sewage outlets that also receive hospital wastewater in Vellore, Tamil Nadu, India around 9:00–11:00 a.m. on 25th May 2023. Geographical coordinates of sampling sites are provided in Table A1. The sampling sites were strategically chosen due to the presence of several hospitals in the surrounding area, with the rationale that hospital effluents are likely contributors to the environmental burden of ARB, thus facilitating the assessment of their resistance profiles. Four sewage samples (each 500 mL) were collected and transferred to a sterile amber glass reagent bottle and the pH was checked on site. The samples were immediately brought to the laboratory for isolation and stored at −20°C for further analysis.

### 2.3 Sample analysis

Liquid-liquid extraction of the sewage samples was performed and the extract was filtered through 0.22 μm sterile filter. The LC/MS filtered samples were inserted into LC/MS system to detect the presence of antibiotics. All four sewage samples were analyzed via LC/MS (Sample 1 by Thermo Scientific TSQ Quantis plus LCMS/MS in ESI+ mode, and sample 2, 3 and 4 by Agilent 6460 Triple Quad 6460, ESI+ mode). The in-house method used for the sample preparation is discussed below.

The sewage samples were extracted via the liquid-liquid extraction method. EDTA salt (0.5 g) was added to 10 ml of each sample, and the mixture was shaken well for 2 min. Next, the samples were sonicated for 1 min in an ultrasonic water bath and vortexed for 2 min. The sample solution with 20 ml of dichloromethane (DCM) was transferred into a separating funnel and shaken well for 5 min. Later, the DCM layer was collected, and the process was repeated by adding another 20 ml of DCM. The collected DCM layer was passed through sodium sulfate in a glass funnel to absorb any remaining moisture. The extracted DCM solution was subsequently subjected to nitrogen evaporation to dryness. The dried extract was reconstituted with 2 ml of methanol and water (20:80) and filtered through a 0.22 μm nylon syringe filter. The same extraction method was followed for antibiotic standards as well. The samples and standards were then injected into LC/MS system.

### 2.4 Isolation and purification of antibiotic-resistant bacteria

The collected sewage samples were immediately subjected to serial dilution (up to 10^−6^), and 100 μL of each diluted sample was spread onto minimal salt medium (MSM) agar plates (composition: 2 g/L NH_4_Cl, 1.5 g/L K_2_HPO_4_, 0.5 g/L KH_2_PO_4_, 0.2 g/L MgSO_4_.7H_2_O, 0.2g/L CaCl_2_.2H_2_O, 0.02 g/L FeCl_3_.6H_2_O, 0.02 g/L ZnCl_2_ and 0.002g/L Na_2_MoO_4_, 5 g/L dextrose) supplemented with 10 μg/mL of amoxicillin trihydrate, meropenem trihydrate and vancomycin hydrochloride. The plates were then incubated at 37°C for 24–48 h (Cavalcante and Dobereiner, 1988; Hartmann and Baldani, 2006; Yan et al., 2022).

For the enrichment-based isolation method, 5% sewage was added to MSM supplemented with 10 μg/mL of amoxicillin trihydrate, meropenem trihydrate, and vancomycin hydrochloride. The broth was incubated under shaking conditions at 120 rpm and 37°C for 4 days. Following incubation, 4 mL of the broth culture was transferred to a fresh, sterilized MSM supplemented with 20 μg/mL amoxicillin trihydrate, meropenem trihydrate, and vancomycin hydrochloride. The final volume of the medium was adjusted to 100 mL, and the culture was incubated under identical conditions as previously described for another 4 days. Subsequently, 6 mL of the culture was inoculated into fresh sterilized MSM containing 40 μg/mL amoxicillin trihydrate, meropenem trihydrate, and vancomycin hydrochloride, with the final medium volume adjusted to 100 mL. The culture was incubated as described above. After 4 days, 8 mL of the culture was transferred to fresh sterilized MSM supplemented with 60 μg/mL amoxicillin trihydrate, meropenem trihydrate, and vancomycin hydrochloride, maintaining a final volume of 100 mL, and incubated under the same conditions for 4 days. This process was repeated with sequential inoculations, 10 mL of culture into MSM containing 80 μg/mL amoxicillin trihydrate, meropenem trihydrate, and vancomycin hydrochloride, and finally 12 mL of culture into MSM with 100 μg/mL amoxicillin trihydrate, meropenem trihydrate, and vancomycin hydrochloride. In each case, the final medium volume was adjusted to 100 mL, and the cultures were incubated for 4 days in shaking conditions at 120 rpm and 37°C. The isolation of antibiotic resistant bacteria from enriched samples was carried out by taking 1 mL of sample and subjecting it to serial dilution (up to 10^−6^) and 100 μL of each diluted sample was spread on to MSM agar plates supplemented with 10 μg/mL amoxicillin trihydrate, meropenem trihydrate, and vancomycin hydrochloride (Yang et al., 2019).

The morphologically distinct colonies were picked and purified on MSM agar plates. The purified cultures were also sub-cultured on Mueller-Hinton Agar (MHA) and stored in 50% glycerol solution at −80°C for long-term storage (Stephen and Saleh, 2023).

### 2.5 Morphological and biochemical characterization

The purified bacterial isolates were subjected to morphological and biochemical characterization. Initial identification tests involved Gram staining, assessment of colony morphology, and conventional biochemical assays including catalase and oxidase activity tests. For further biochemical profiling all the isolates were analyzed via the commercial KB002 HiAssorted^TM^ Biochemical Test Kit (HiMedia Laboratories Ltd., Mumbai, India) which comprises a panel of 12 standard biochemical tests for the identification of gram-negative rods. The tests include citrate utilization test, lysine utilization, ornithine utilization, urease detection, phenylalanine deamination (TDA), nitrate reduction, H_2_S production test, and 5 different carbohydrate utilization tests for glucose, adonitol, lactose, arabinose, and sorbitol (Prabhukarthikeyan et al., 2015; Sousa et al., 2015).

### 2.6 Phenotypic virulence determination

The phenotypic virulence of the isolates was determined via hemolytic, lipolytic, amylase, protease, and biofilm formation by Congo red agar tests.

#### 2.6.1 Hemolytic test

Overnight grown cultures were inoculated in nutrient agar (NA) media supplemented with 5% sheep blood. The plates were incubated at 30°C for 24 h. A clear zone around the inoculum suggests the blood lysis capability of bacteria by the production of hemolysin, and the absence of a zone around the inoculum suggests their non-hemolytic capacity (Ghosh et al., 2024).

#### 2.6.2 Lipolytic test

The lipase production test was confirmed by inoculating overnight-grown cultures in Tween-80 medium for 24 h at 37°C. A clear zone around bacterial growth indicates the production of lipase by the bacteria (Ghosh et al., 2024).

#### 2.6.3 Amylase test

The production of amylase by the bacteria was confirmed by inoculating the overnight grown culture onto MHA media and incubating it for 24 h at 37°C. The bacteria producing amylase forms a clear zone around the growth (Lee, 1976).

#### 2.6.4 Protease test

The production of protease by the isolates was confirmed by inoculating an overnight seed culture of each isolate in NA supplemented with 1.5% (w/v) of skim milk agar. The plates were incubated for 24 h at 37°C. The isolate that able to degrade casein presents a clear zone around the area of bacterial growth (Ghosh et al., 2024).

#### 2.6.5 Biofilm formation by the Congo red agar assay

The Congo red agar (CRA) method for the biofilm formation assay was followed as described by (Freeman et al. 1989). In this method, CRA was prepared via the use of brain heart infusion (BHI) broth (37 g/L), 5% sucrose, 1% agar and 0.08% Congo red. Congo red was prepared as a concentrated aqueous solution and sterilized separately from the other media. Later, both solutions were mixed such that agar cooled to 55°C. Once the prepared plates were cooled, fresh cultures were inoculated and incubated for 24 h at 37°C. The appearance of black colonies with dry crystalline consistency indicates the production of biofilms. Pink colonies that sometimes have a slightly darker center are considered as non-biofilm producers. Dark colonies without a dry crystalline colony morphology are indeterminate.

### 2.7 Determination of the minimum inhibitory concentration

The minimum inhibitory concentration (MIC) is the gold standard for assessing bacterial susceptibility and represented the lowest concentration of an antimicrobial agent necessary to inhibit bacterial growth under *in vitro* conditions. The minimum inhibitory concentration (MIC) of bacteria was determined via commercial antibiotic gradient strips and broth microdilution method (Kaderábková et al., 2024). The MIC assay was performed based on EUCAST guidelines.

#### 2.7.1 MIC determination via antibiotic gradient strips

The MIC assay was carried out via commercial antibiotic gradient strips. The amoxicillin and vancomycin (concentrations ranging from 0.016 to 256 μg/mL), and meropenem (concentrations ranging from 0.002 to 32 μg/mL) strips were purchased from HiMedia, Mumbai, India. The pure cultures of bacteria at OD_600_ of 0.08–0.1 were swabbed three times at a 120° angle in MHA plates and allowed to air dry for 5–10 min. Commercially available antibiotic strips were subsequently placed on the agar via the sticky applicator provided with the strips. The plates were incubated upside down for 16–24 h at 37°C. After incubation, bacterial growth was assessed for each antibiotic, and the MIC value was determined (EUCAST, 2003; Kaderábková et al., 2024).

#### 2.7.2 MIC determination via broth microdilution method

For this method, fresh stock solutions of amoxicillin trihydrate, vancomycin hydrochloride, and meropenem trihydrate at an initial concentration of 4,096 μg/mL were prepared using 0.1 M phosphate buffer (pH 6) for amoxicillin, distilled water for vancomycin, and 0.01 M phosphate buffer (pH 7.2) for meropenem. The test was conducted in 96-well plates, with each well having a capacity of 300 μL. The working concentrations for amoxicillin and vancomycin ranged from 0.125 to 2,048 μg/mL, doubling sequentially, whereas for meropenem, the range started from 0.016 to 256 μg/mL, also following a twofold serial dilution pattern. Equal volumes (100 μL of each) of a pure bacterial culture (OD600 = 0.08–0.1) and the respective antibiotic working solutions were added to each well. The plates were incubated at 37°C for 16–24 h. After incubation, visible bacterial growth was assessed by comparing the presence of distinct buttons or turbidity in the inoculated wells against the absence of growth in the uninoculated wells. The MIC was determined as the lowest antibiotic concentration at which no visible growth of bacteria was observed with the naked eye (EUCAST, 2003; Kaderábková et al., 2024).

### 2.8 Molecular identification

The culture was resuspended in 1 mL DNAiso reagent (Takara Bio) and mixed thoroughly by pipetting 8–10 times to ensure complete dispersion. The sample was incubated at room temperature for 5 min, followed by centrifugation at 10,000 rpm for 10 min. The clear supernatant was transferred to a fresh tube and the pellet was discarded. Subsequently, 0.5 mL of absolute ethanol was added to the supernatant, followed by gentle inversion (10–15 times) to facilitate DNA precipitation. The mixture was incubated at room temperature for 3 min, and centrifuged at 10,000 rpm for 10 min to collect the DNA pellet. The supernatant was discarded, and the DNA pellet was washed twice with 500 μL of 75% ethanol. The pellet was air-dried for 20–30 min at room temperature and subsequently dissolved in 30 μL of MilliQ water. DNA quality was confirmed via 1% agarose gel electrophoresis, and the sample was stored at −20°C until further use. The reaction mixture (20 μL) was prepared by adding 1 μL bacterial DNA (10–50 ng), 1 μL each of forward and reverse primers (10 picomoles μL^−1^), 10 μL Emerald Amp GT PCR master mix (Takara Bio) and rest of the volume was made up with nuclease free water. PCR cycling conditions included initial denaturation (94°C for 2 min), cycle denaturation (94°C for 30 s), annealing (55°C for 30 s), extension (72°C for 1.5 min for a total of 30 cycles), and final extension (72°C for 10 min). To confirm the success of PCR, 5 μL of the PCR product was run on 1 % agarose gel containing ethidium bromide at 120 V for 45 min in 1X TAE buffer. A 100 bp (100–1,500 bp) DNA ladder (Takara Bio) was loaded alongside the PCR products as a size marker.

The PCR amplification of the 16S rRNA gene of all the isolates was achieved via the 27F (5′-AGAGTTTGATC(AC)TGGCTCAG-3′) and 1492R (5′-GGTTACCTTGTTACGACTT-3′) primers using BDT v3.1 Cycle sequencing kit on ABI 3730xl Genetic Analyzer. The consensus sequence of the 16S rRNA gene was generated from forward and reverse sequence data via the aligner software. The 16S rRNA gene sequence was used to carry out BLAST with the “nr” database of the NCBI GenBank database. On the basis of the maximum identity score, 20 sequences were selected and aligned using multiple alignment software program Clustal W in MEGA 11 for the construction of a phylogenetic tree via the neighbor joining method (Punom et al., 2016; Bertolo et al., 2024).

### 2.9 Statistical analysis

All the experiments performed in this study were in triplicates. The results for MIC were expressed as MIC log_2_ mean value, subsequently standard deviation (SD), and standard error (SE) calculated using Microsoft excel. Significant differences among the different antibiotic supplementation across species were calculated using two-factor ANOVA. Paired *t*-test was conducted to check the compatibility of two MIC methods.

## 3 Results

### 3.1 Sample analysis

Four sewage samples were collected and the pH was measured. The pH values of the sewage sample 1, sample 2, sample 3 and sample 4 were 8, 9, 9, and 7.78, respectively. The collected sewage samples were analyzed for the presence of antibiotics including amoxicillin, meropenem, and vancomycin (μg/L) by LC/MS. The limit of quantification (LOQ) was 0.01 for amoxicillin, meropenem, and vancomycin. All three antibiotics were found to be below the limit of quantification (BLQ) in the sewage sample (Table 2). The TIC MRM of all the sewage samples and antibiotic standards are provided in the additional file (Figures A1–A6).

**Table 2:** LC/MS result of antibiotics in the analyzed sewage samples.

**Sl. No**.	**Antibiotics**	**Unit**	**Sample 1**	**Sample 2**	**Sample 3**	**Sample 4**	**LOQ**
1	Amoxicillin	μg/L	BLQ	BLQ	BLQ	BLQ	0.01
2	Meropenem	μg/L	BLQ	BLQ	BLQ	BLQ	0.01
3	Vancomycin	μg/L	BLQ	BLQ	BLQ	BLQ	0.01

BLQ, Below limit of quantification; LOQ, Limit of quantification.

### 3.2 Isolation and purification of antibiotic-resistant bacteria

**Figure 1:**
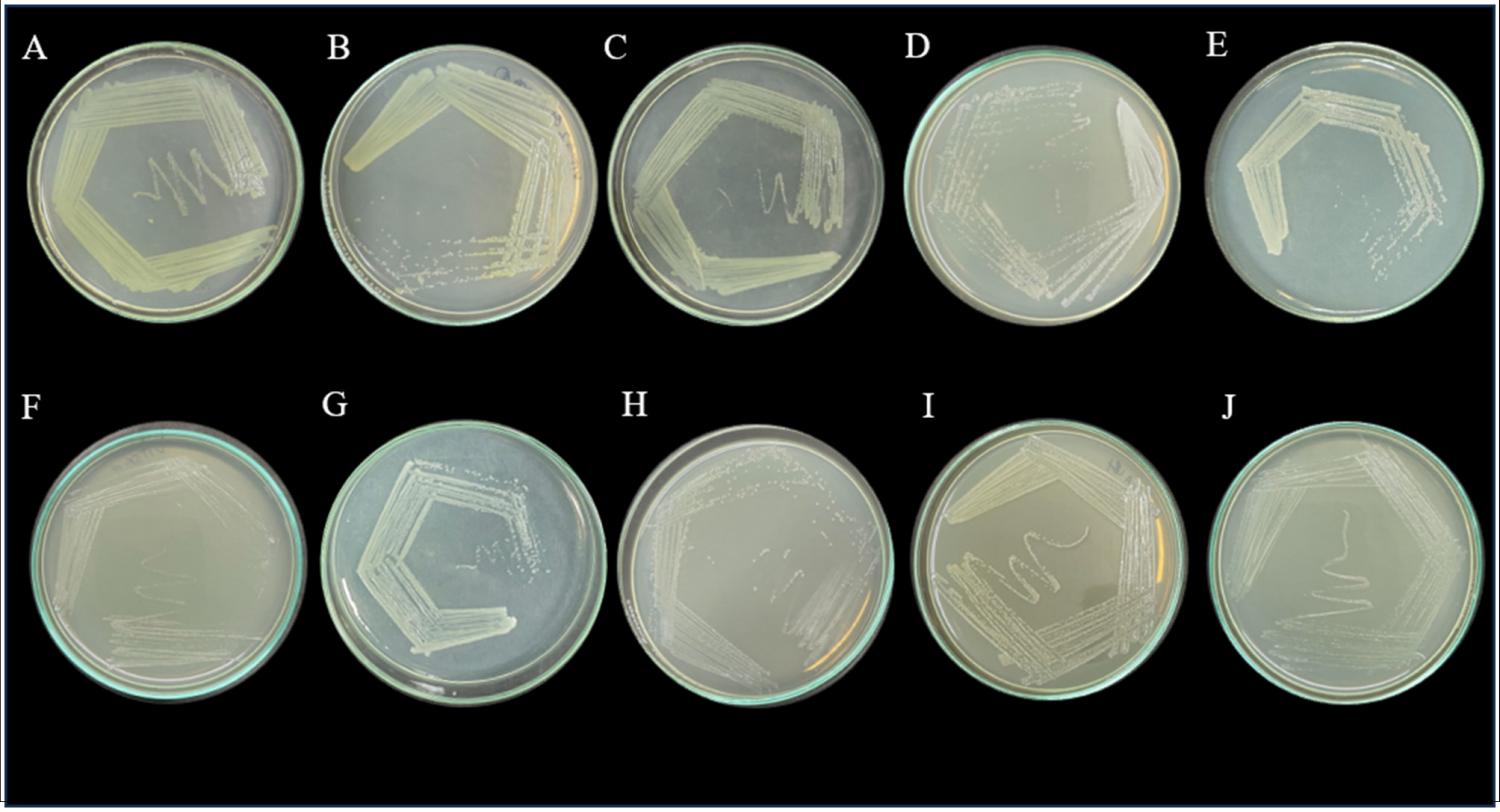
Purified bacterial strains on Mueller-Hinton agar plates- **(A)** VITSA1, **(B)** VITSA2, **(C)** VITSA3, **(D)** VITSA4, **(E)** VITSA8, **(F)** VITSA11, **(G)** VITSA12, **(H)** VITSA13, **(I)** VITSA14, **(J)** VITSA19.

A total of 21 microorganisms were isolated from all the sewage samples on MSM media supplemented with amoxicillin trihydrate, meropenem trihydrate, and vancomycin hydrochloride (10 μg/mL). These microbial isolates were further purified on MHA. Among the 21 strains, 10 were bacterial isolates and 11 were yeasts, which were confirmed with a compound microscope. The 10 bacterial isolates were further purified and maintained on MHA (Figure 1). These bacterial isolates were further characterized morphologically and biochemically. The strains were screened for their phenotypic virulence traits and antibiotic resistance properties.

### 3.3 Morphological and biochemical characterization

A set of biochemical tests were carried out to identify the isolated bacteria via the HiAssorted^TM^ Biochemical Test Kit. All the bacterial isolates were rod-shaped, gram negative, and catalase positive. Six isolates tested oxidase-positive while four were oxidase-negative. The colony characteristics and microscopic observations of the bacterial isolates are given in Table 3. The results for all the isolates were compared with the HiMedia standard reference table provided with the kit. The results of VITSA14 were highly similar with those to *Klebsiella* species. The other 9 isolates did not have any similarity with any of the species provided in the kit. None of the isolates produced H_2_S gas. In addition, only VITSA3 was able to produce urease enzyme. The details of the biochemical test results are provided in Table 4.

**Table 3:** Microscopic observations and colony characteristics of all the bacterial isolates.

	**Microscopic observations**		**Colony characteristics**				
**Isolates**	**Gram's stain retention**	**Shape**	**Colony form**	**Colony margin**	**Colony texture**	**Colony size**	**Colony color**
VITSA1	Gram negative	Rod	Circular	Entire	Smooth	Medium	Light yellow
VITSA2	Gram negative	Rod	Circular	Entire	Smooth	Medium	Light yellow
VITSA3	Gram negative	Rod	Circular	Entire	Smooth	Small	Cream
VITSA4	Gram negative	Rod	Circular	Entire	Smooth	Small	White
VITSA8	Gram negative	Rod	Circular	Entire	Smooth	Small	Cream
VITSA11	Gram negative	Rod	Circular	Entire	Smooth	Pin point	Cream
VITSA12	Gram negative	Rod	Circular	Entire	Smooth	Small	Cream
VITSA13	Gram negative	Rod	Circular	Entire	Smooth	Medium	White
VITSA14	Gram negative	Rod	Circular	Entire	Smooth	Big	White
VITSA19	Gram negative	Rod	Circular	Entire	Smooth	Pin point	Cream

**Table 4:** Biochemical characteristics of all the bacterial isolates.

**Isolates**	**Catalase**	**Oxidase**	**Citrate utilization**	**Lysine**	**Ornithine**	**Urease**	**TDA**	**Nitrate reduction**	**H_2_S production**	**Glucose**	**Adonitol**	**Lactose**	**Arabinose**	**Sorbitol**
VITSA1	+	–	+	+	+	+	–	+	–	–	–	–	–	–
VITSA2	+	–	+	+	+	+	–	+	–	–	–	–	–	–
VITSA3	+	+	–	–	–	–	+	–	–	–	–	–	–	–
VITSA4	+	+	+	+	+	+	–	+	–	–	–	–	–	–
VITSA8	+	+	+	+	+	+	–	+	–	–	–	–	–	–
VITSA11	+	+	+	–	+	+	–	–	–	–	–	–	–	–
VITSA12	+	+	–	+	+	+	–	+	–	–	–	–	–	–
VITSA13	+	–	–	+	+	+	–	–	–	–	–	–	–	–
VITSA14	+	–	+	+	–	+	–	+	–	+	+	+	+	+
VITSA19	+	+	+	+	+	+	–	+	–	–	–	–	–	–

+, Positive (more than 90%); –, Negative (more than 90%).

### 3.4 Phenotypic virulence determination

**Figure 2:**
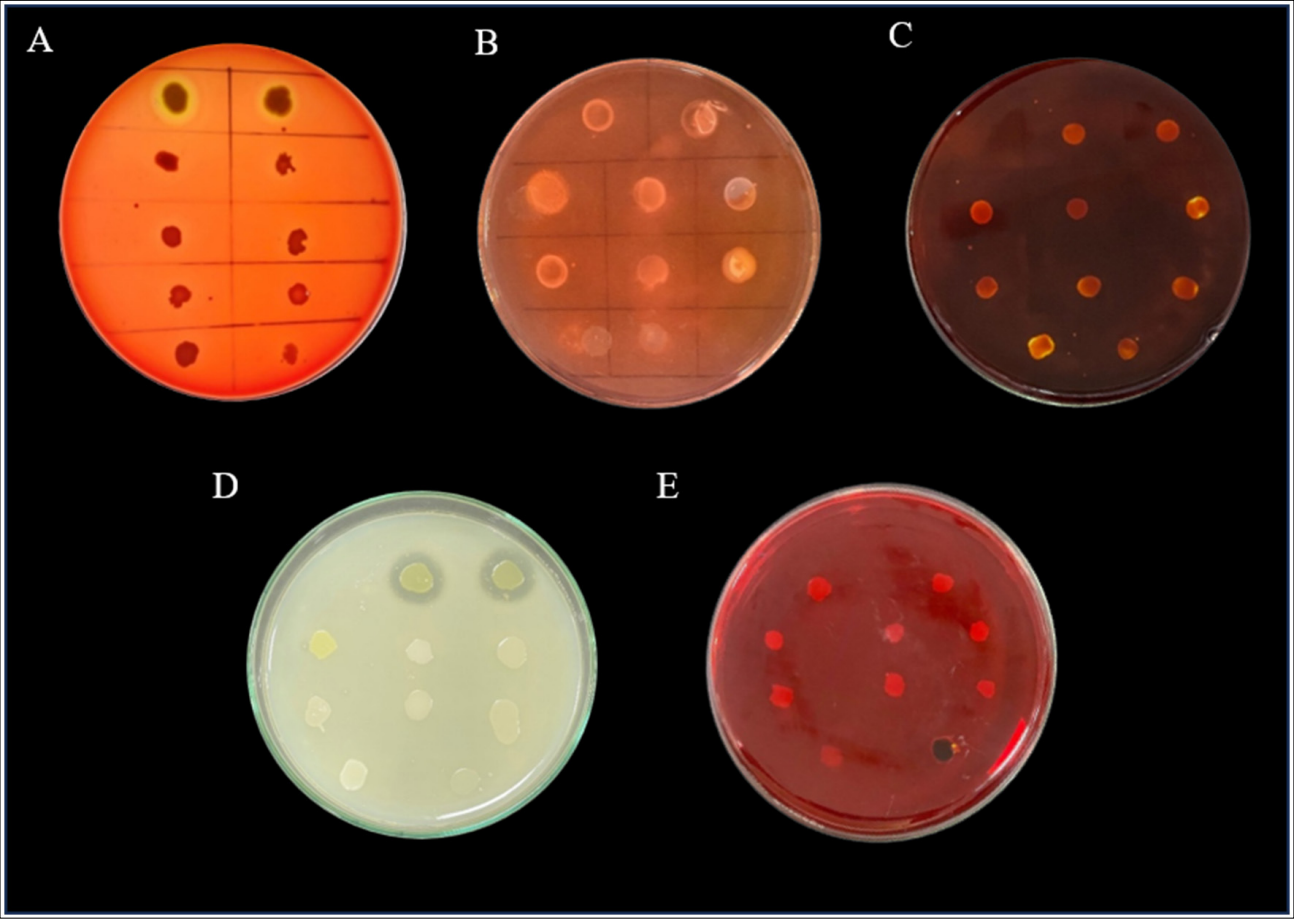
Phenotypic virulence tests of bacterial isolates- **(A)** hemolytic test, **(B)** lipase test, **(C)** amylase test, **(D)** protease test, and **(E)** Congo red agar tests. All the 10 bacterial isolates inoculated in single plate- (a) VITSA1, (b) VITSA2, (c) VITSA3, (d) VITSA4, (e) VITSA8, (f) VITSA11, (g) VITSA12, (h) VITSA13, (i) VITSA14, and (j) VITSA19.

Phenotypic virulence tests such as hemolytic test, lipase, amylase, protease, and biofilm formation assay by Congo red agar test were performed. Only two of the 10 bacteria (VITSA1 and VITSA2) were found to be hemolytic in blood agar plates (qualitative test). None of the isolates produced amylase or lipase; however, VITSA1 and VITSA2 showed protease activity. Congo red agar test revealed that all the cultures were in the range from white to pink in color with no dark center and the absence of dried crystalline colonies which indicated that the isolates were non-biofilm producers. VITSA14 appeared black but had smooth colonies rather than dried crystalline colonies which indicated indeterminant results. The results of all the tests are shown in Figure 2.

### 3.5 Determination of the minimum inhibitory concentration

**Figure 3:**
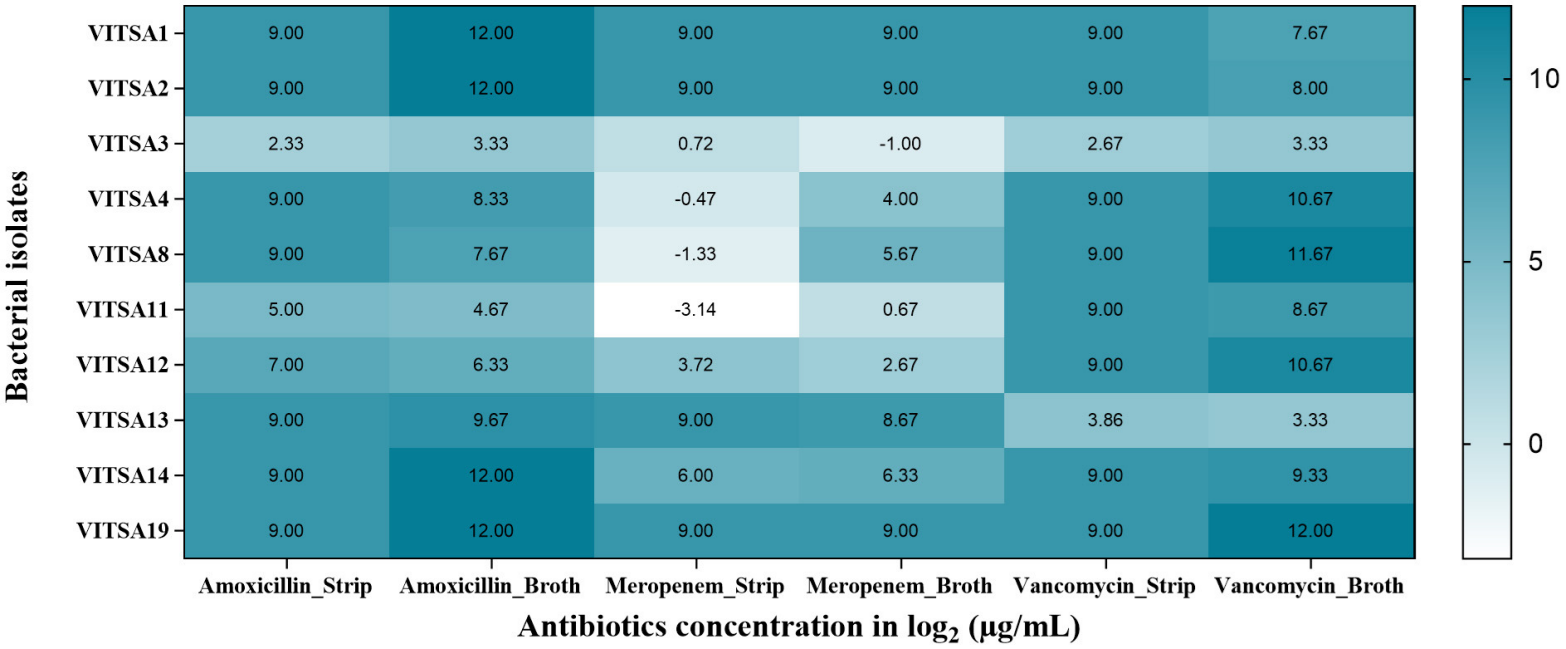
Heat map representing the mean MIC values (log_2_ μg/mL) of each bacterial isolate against three antibiotics (amoxicillin, meropenem, and vancomycin) measured using gradient strip method (Amoxicillin_strip, Meropenem_Strip, Vancomycin_Strip) and broth microdilution method (Amoxicillin_Broth, Meropenem_Broth, Vancomycin_Broth). Values represent the average of three independent measurements (log_2_ μg/mL) for each isolate (darker shades of blue indicate higher resistance).

**Figure 4:**
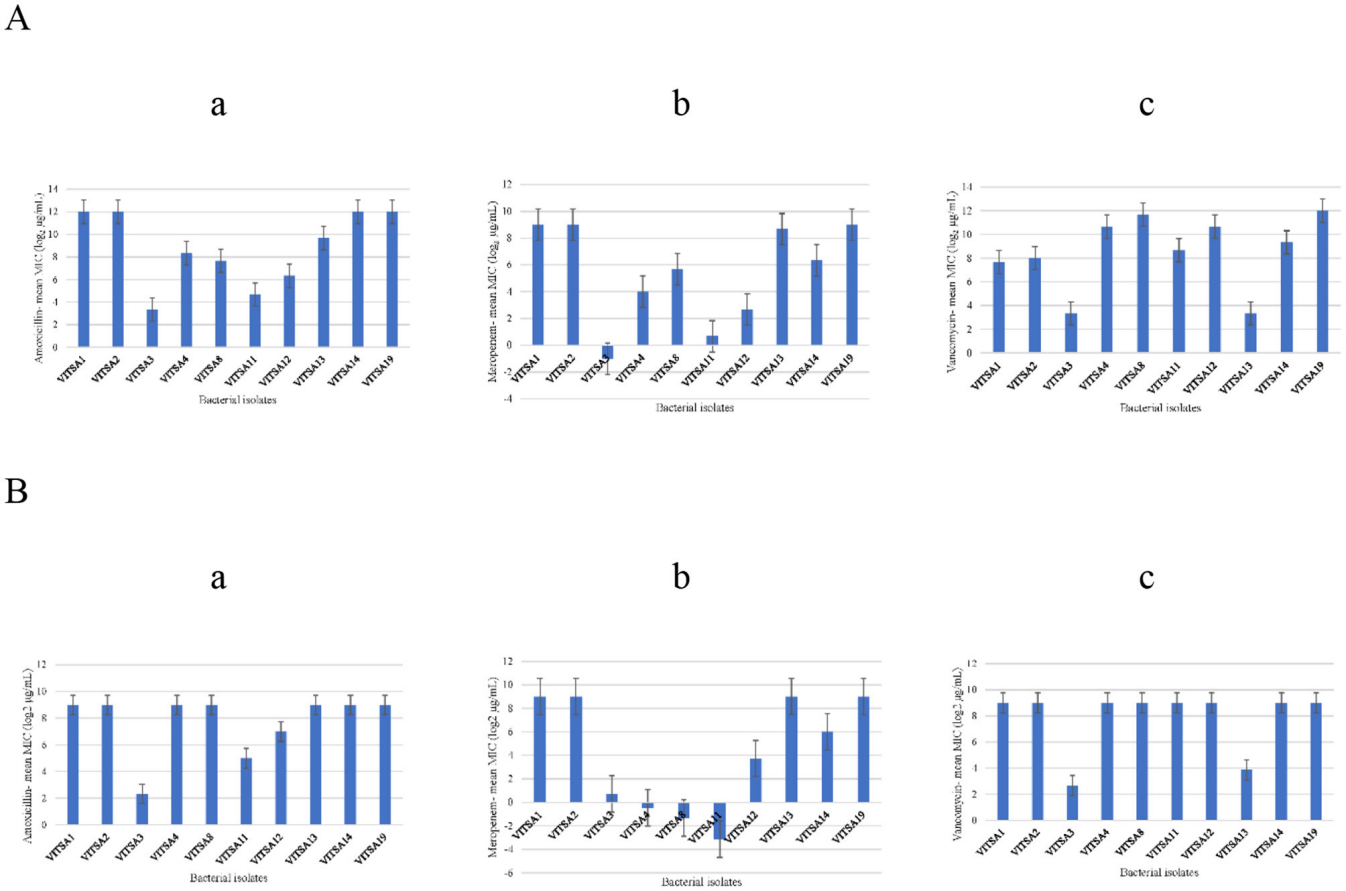
Minimum inhibitory concentration (MIC) values (log_2_ μg/mL) of bacterial isolates against three tested antibiotics determined by two methods. **(A)** Mean MIC values (log_2_ μg/mL) obtained using the broth microdilution method: (a) amoxicillin, (b) meropenem, and (c) vancomycin. **(B)** Mean MIC values (log_2_ μg/mL) obtained using the antibiotic gradient strips method: (a) amoxicillin, (b) meropenem, and (c) vancomycin.

The MICs of amoxicillin, meropenem, and vancomycin were determined for all 10 bacterial isolates using the gradient strip and broth microdilution methods. The MIC values with triplicates for all three tested antibiotics is provided in Tables A2, A3. The MIC gradient strips results are provided in Figure A8. The MIC values were log_2_-transformed (μg/mL) for comparative and statistical analyses. The results are summarized in Tables A4, A5, with visual representation in Figure 3 (heat map) and Figure 4 (bar graphs), and correlation plots in Figure A7 (scatter plots). The gradient strip method revealed that strains VITSA1, VITSA2, and VITSA19 had MIC values ≥ 512 μg/mL for all three tested antibiotics. In contrast, the broth microdilution method showed even higher resistance, with amoxicillin MICs ≥ 4,096 μg/mL for VITSA1, VITSA2, VITSA14, and VITSA19, and with vancomycin MICs 256- ≥ 4,096 for VITSA4, VITSA8, VITSA12, and VITSA19. Notably, meropenem resistance was consistently high (≥512 μg/mL) across VITSA1, VITSA2, VITSA13, VITSA14, and VITSA19 by both methods. These extremely high MIC values suggest strong resistance phenotypes, particularly against β-lactams and glycopeptides. Mean MIC values (log_2_-transformed) were calculated from triplicate measurements and compared between methods using paired *t*-tests (Table A6) and scatter plots (Figure A7). Despite some variability in individual isolate values, the paired *t*-test showed no statistically significant difference between the two methods for any antibiotic (*p* > 0.05). Additionally, a two-factor ANOVA conducted using log_2_ MIC values revealed a significant difference in resistance patterns among isolates and antibiotics (*p* < 0.05), confirming method-independent variability in antimicrobial susceptibility. The heat map (Figure 3) further highlights the diversity in resistance profiles among isolates across both methods. For instance, VITSA3 displayed moderate susceptibility (log_2_ MIC = −1 to 3), while VITSA1, VITSA2, and VITSA19 showed consistent and extreme resistance (log_2_ MIC ≥ 7) across all the tested antibiotics. These findings validate the presence of multidrug resistance in several environmental isolates and demonstrate the robustness of combing broth and strip-based MIC testing with statistical approaches for resistance profiling.

### 3.6 Molecular identification

**Figure 5:**
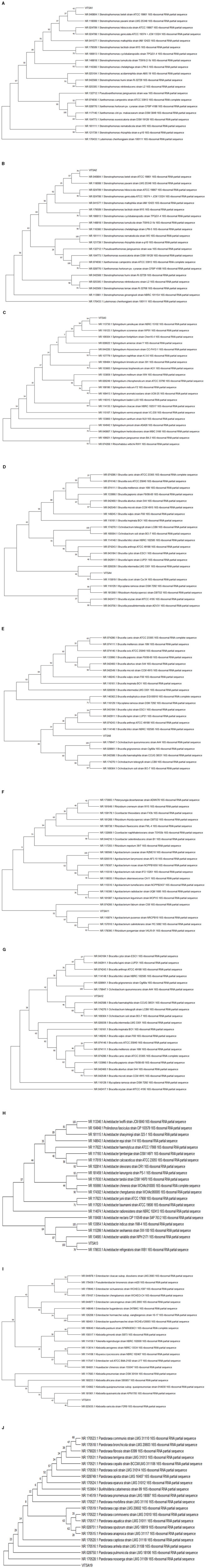
Phylogenetic tree of the bacterial isolates- **(A)** VITSA1, **(B)** VITSA2, **(C)** VITSA3, **(D)** VITSA4, **(E)** VITSA8, **(F)** VITSA11, **(G)** VITSA12, **(H)** VITSA13, **(I)** VITSA14, **(J)** VITSA19.

The phylogenetic tree was constructed by the neighbor-joining method using the sequences of 16S rRNA gene. Mega 11 software was used to construct phylogenetic trees. VITSA1 had the highest 16S rRNA gene sequence similarity with *Stenotrophomonas maltophilia* strain IAM 12423 (99.4%), VITSA2 with *Stenotrophomonas pavanii* strain LMG 25348 (99.66%), VITSA3 with *Sphingobium yanoikuyae* strain NBRC 15102 (99.72%), VITSA4 with *Brucella intermedia* LMG 3301 (99.52%), VITSA8 with *Brucella anthropi* ATCC 49188 (96.95%), VITSA11 with *Agrobacterium radiobacter* strain UQM 1685 (99.10%), VITSA12 with *Ochrobactrum teleogrylli* strain LCB8 (98.27%), VITSA13 with *Acinetobacter refrigeratoris* strain WB1 (99.13%), VITSA14 with *Klebsiella pneumoniae* strain DSM 30104 (99.73%) and VITSA19 with *Pandoraea fibrosis* strain 6399 (99.22%). The 16S rRNA gene sequences of all 10 bacterial strains were submitted to the NCBI database, and their GenBank accession numbers along with their percentage identities with the most similar bacterial strains are listed in the Table 5. The phylogenetic tree of all bacterial isolates is provided in Figure 5.

**Table 5:** Molecular identification and accession numbers of all the bacterial isolates.

**Isolate**	**Closest NCBI GenBank relative**	**Accession no. of closest NCBI GenBank relative**	**Length (bp)**	**% Identity**	**NCBI GenBank accession no**.
VITSA1	*Stenotrophomonas maltophilia* strain IAM 12423 16S ribosomal RNA, partial sequence	NR_041577.1	1,507	99.40%	PV421299
VITSA2	*Stenotrophomonas pavanii* strain LMG 25348 16S ribosomal RNA, partial sequence	NR_118008.1	1,499	99.66%	PV421304
VITSA3	*Sphingobium yanoikuyae* strain NBRC 15102 16S ribosomal RNA, partial sequence	NR_113730.1	1,440	99.72%	PV421305
VITSA4	*Brucella intermedia* LMG 3301 16S ribosomal RNA, partial sequence	NR_026039.1	1,449	99.52%	PV421306
VITSA8	*Brucella anthropi* ATCC 49188 16S ribosomal RNA, partial sequence	NR_043184.1	1,406	96.95%	PV421364
VITSA11	*Agrobacterium radiobacter* strain UQM 1685 16S ribosomal RNA, partial sequence	NR_116306.1	1,450	99.10%	PV533977
VITSA12	*Ochrobactrum teleogrylli* strain LCB8 16S ribosomal RNA, partial sequence	NR_174270.1	1,447	98.27%	PV533985
VITSA13	*Acinetobacter refrigeratoris* strain WB1 16S ribosomal RNA, partial sequence	NR_178633.1	1,498	99.13%	PV533987
VITSA14	*Klebsiella pneumoniae* strain DSM 30104 16S ribosomal RNA, partial sequence	NR_117683.1	1,521	99.73%	PV533988
VITSA19	*Pandoraea fibrosis* strain 6399 16S ribosomal RNA, partial sequence	NR_179020.1	1,529	99.22%	PV533990

## 4 Discussion

The escalating global threat of antimicrobial resistance (AMR) is increasingly recognized as a critical One Health challenge. Environmental reservoirs such as wastewater systems play a pivotal role in the dissemination of resistant bacteria (Salam et al., 2023). The presence of antibiotic in the sampled sewage may be contributing to sub-inhibitory selective pressure, fostering horizontal gene transfer and the proliferation of resistant strains (Flores-Vargas et al., 2021). Sewage serves as a hotspot of AMR due to prolonged exposure to sub-inhibitory concentrations of antibiotics (Fouz et al., 2020; Flores-Vargas et al., 2021). Therefore, sewage is recognized as a critical reservoir for ARB and ARGs globally (Fouz et al., 2020). In the present study, the sewage samples were investigated for the occurrence of antibiotics and the LC/MS analysis of sewage samples indicated that the concentration of all the tested antibiotics was BLQ. For instance, amoxicillin is the most frequently detected antibiotic in wastewater due to its high excretion rate which is upto 80% within 2 h of ingestion (Aryee et al., 2022). The reported concentrations of amoxicillin ranges from 33,800 ng/L to over 116,400 ng/L in raw and treated wastewater (Mutuku et al., 2022). This widespread presence of antibiotics contributes to selective pressures that promote the persistence and evolution of MDR strains. This is consistent with previous studies indicating that environmental exposure to low antibiotic concentration can drive AMR development (Flores-Vargas et al., 2021).

In this study, sewage samples collected from regions influenced by hospital effluents revealed the presence of diverse bacterial species exhibiting high levels of antibiotic resistance against the antibiotics including amoxicillin, meropenem, and vancomycin. We have identified 10 isolates belonging to genus *Stenotrophomonas, Sphingobium, Brucella, Agrobacterium, Ochrobactrum, Acinetobacter, Klebsiella*, and *Pandoraea* while (Mehanni et al. 2023) have reported *Escherichia, Enterococcus*, and *Staphylococcus* spp. from hospital effluent resistant to multiple antibiotics, including amoxicillin. The most significant resistance observed in the present study was against amoxicillin, with the four identified isolates, including *Stenotrophomonas* sp. strain VITSA1, *S. pavanii* strain VITSA2, *K*. *pneumoniae* strain VISA14, and *Pandoraea* sp. strain VITSA19, showing MIC values ≥ 4,096 μg/mL. This MIC value was far exceeding the therapeutic thresholds. The resistance by *Stenotrophomonas* sp. strain VITSA1, *S. pavanii* strain VITSA2, *Acinetobacter* sp. strain VITSA13, and *Pandoraea* sp. strain VITSA19 to meropenem was around 256– ≥ 512 μg/mL. *Stenotrophomonas* sp. strain VITSA1, *S. pavanii* strain VITSA2, *Brucella* spp. strains VITSA4 and VITSA8, *A. radiobacter* strain VITSA11 and *Ochrobactrum* sp. strain VITSA12, *K*. *pneumoniae* strain VISA14 and *Pandoraea* sp. strain VITSA19 were found to resistance to vancomycin with MIC values in the range 256– ≥ 4,096 μg/mL. This higher resistance expands the spectrum of environmental bacteria contributing to AMR.

Species-specific observations further highlight the environmental AMR landscape. EUCAST (European Committee on Antimicrobial Susceptibility Testing) and CLSI (Clinical and Laboratory Standards Institute) use breakpoints (specific antibiotic concentrations) to categorize a bacterial as susceptible, intermediate or resistant to that antibiotic (ESCMID^1^; CLSI, 2020). *S. maltophilia* is reported to be resistant to amoxicillin and meropenem by CLSI (2020). Likewise, we have reported *Stenotrophomonas* sp. VITSA1 as resistant to amoxicillin and meropenem, and additionally to vancomycin as well with high MIC values. *S. yanoikuyae*, though commonly associated with environmental functions such as bioremediation (Cunliffe and Kertesz, 2006), exhibited resistance profiles of concern. The resistant breakpoints for amoxicillin, meropenem, and vancomycin of *S. pavanii, S. yanoikuyae, Brucella* spp., *Agrobacterium* spp., *Ochrobactrum*. spp., and *Pandoraea* spp. are not available on the CLSI and EUCAST guidelines. According to the CLSI and EUCAST, *Acinetobacter* spp. lacks relevant resistant breakpoints for amoxicillin and vancomycin, although meropenem resistant breakpoint is ≥ 8 μg/mL (see text footnote 1; CLSI, 2020). Findings of this study shows that *Acinetobacter* sp. strain VITSA13 exceeded the resistant breakpoint value (≥8 μg/mL) for meropenem set by CLSI and EUCAST, with MIC reaching around 256–512 μg/mL. This result indicates a potential clinical treatment failure in the event of an infection with resistant *Acinetobacter* spp. The MIC QC ranges of *K. pneumoniae* ATCC 700603 for amoxicillin is >128 μg/mL, of *K. pneumoniae* ATCC BAA-1705 for meropenem is 8–64 μg/mL and of *K. pneumoniae* ATCC BAA-2814 for meropenem is 32–256 μg/mL (CLSI, 2020). However, the resistance shown by *Klebsiella pneumoniae* strain VITSA14 against meropenem (MIC value of 64 μg/mL), a last-resort antibiotic for treating severe bacterial infections, is concerning. *K. pneumoniae* strain VITSA14 displayed high resistance to amoxicillin and meropenem, which aligns with previous reports indicating the presence of extended-spectrum beta-lactamase (ESBL) and carbapenemase genes such as *bla*_*OXA*−48_, *bla*_*KPC*_, *bla*_*NDM*_, *bla*_*IMP*_, *bla*_*VIM*_, *bla*_*SHV*−1_, *bla*_*TEM*−1*A*_, *bla*_*CTX*−*M*−15_, and *bla*_*VEB*−1_ (Sharma et al., 2023; Karaman et al., 2024). The extreme resistance observed in *Pandoraea* sp. VITSA19 is particularly alarming, with MIC values reaching ≥ 512 μg/mL for meropenem, ≥ 4,096 μg/mL for vancomycin, and ≥ 4,096 μg/mL for amoxicillin.

The study highlights the growing concern of multidrug resistance among environmental bacterial species., with several strains exhibiting elevated MIC values and resistance to critical antibiotics like vancomycin, meropenem, and amoxicillin. Several studies have demonstarted that *S. maltophilia* and *S. pavanii* are resistant to beta-lactams and vancomycin, and harbor multidrug resistance genes, including *aac(6*′*)-Iak* (Gülmez and Hasçelik, 2005; Boedeker et al., 2010; Tada et al., 2014; Fluit et al., 2022). The presence of genes encoding multidrug resistance efflux pumps and antibiotic inactivation enzymes in *S. maltophilia* contributes to the development of resistance to many commonly used antibiotics (Gil-Gil et al., 2020). The study found an elevated MIC value for vancomycin (>256 μg/mL) in *S. pavanii* strain VITSA2 which may indicate the development of resistance mechanisms in it. In our study *S. yanoikuyae* strain VITSA3 exhibited moderate resistance to amoxicillin (4 μg/mL), meropenem (0.5 μg/mL), and vancomycin (4 μg/mL), and *S. yanoikuyae* is well known for its capacity to degrade polycyclic aromatic hydrocarbons (Cunliffe and Kertesz, 2006). As this species has degradation role, therefore, its resistance profile necessitates additional research to assess its potential role as an environmental reservoir for antibiotic resistance genes. Previous studies reported resistance levels of *Brucella* spp. up to 16 μg/mL for vancomycin (Mortensen et al., 1986), however, our findings show MIC value exceeding 256 μg/mL. This suggests a significant shift in resistance patterns of *Brucella* spp. The resistance profile of *Acinetobacter* sp. strain VITSA13 was consistent with previous findings, showing a vancomycin MIC value of 96 μg/mL (Namdari et al., 2003). Additionally, (Balmer et al. 2022) suggested that *Agrobacterium* spp. is generally susceptible to carbapenems, however our findings show their MIC value 0.25–32 μg/mL. (Yagel et al. 2020) reported the MIC values for meropenem (0.5–1 μg/mL) and amoxicillin-clavulanic acid (≥32 μg/mL) of *Ochrobactrum* spp. which indicate a slight stable resistance pattern within this genus as our findings reports MIC values for amoxicillin is 64–128 μg/mL and meropenem is 1–16 μg/mL of *Ochrobactrum* sp. strain VITSA12. The findings for *Acinetobacter* sp. strain VITSA13 were also noteworthy, as this species presented an MIC value of 4–16 μg/mL for vancomycin and 256– ≥ 512 μg/mL for meropenem. However, the presence of these resistance levels in clinical and environmental settings require further study.

MIC profiling revealed variable resistance across isolates, with some exhibiting both resistance and virulence traits. The co-occurrence of resistance and virulence in isolates like VITSA1 and VITSA2, which demonstrated hemolysis and protease activity, underscores the potential threat of environmental bacteria. However, *Pandoraea* sp. strain VITSA19 was lacking any detectable virulence traits but showed extreme resistance to all tested antibiotics. The lack of correlation between virulence traits and resistance highlights the complex nature of AMR in the environment. The coexistence of resistance and virulence traits, although evident in certain isolates, was not uniform. This highlights the complexity of AMR evolution in environmental bacteria and the importance of integrating both resistance and pathogenicity assessments in AMR surveillance in the environmental matrices. This study reinforces for the urgent need to raise public awareness. People should be educated to use antibiotics only under medical supervision, complete the prescribed dosage, and avoid purchasing antibiotics without a valid prescription. It also highlights the need for effluent regulations targeting hospitals and pharmaceutical industries. It also emphasizes the importance of advanced wastewater treatment technologies, early warning systems, public health advisories, and metagenomic surveillance of wastewater to monitor antimicrobial resistance.

Despite its important insights, the study had several limitations. Firstly, quantitative virulence assays (e.g., tissue culture plate biofilm method) were not employed, limiting the accuracy of biofilm detection. Secondly, the samples were collected only once from a specific location without accounting for seasonal or spatial variation, which can influence AMR distribution. Thirdly, control samples from uncontaminated or upstream sites were not included, making it difficult to definitely attribute resistance patterns to hospital discharge. However, the detection of high MICs and BLQ levels of antibiotics still strongly points to anthropogenic influence and environmental risk.

This study provides compelling evidence that sewage not only harbors highly resistant environmental bacteria but also serves as a dynamic interface for the environmental amplification and potential clinical re-entry of AMR. Continued investment in environmental AMR research and integrated surveillance strategies is essential to mitigate this growing threat.

## 5 Conclusion

This study provides comprehensive insights into the prevalence and antibiotic resistance profiles of ARB isolated from sewage samples. The high resistance levels observed among various bacterial species highlight the role of sewage as a significant reservoir of ARB and subinhibitory concentrations of antibiotics, contributing to the environmental dissemination of resistance genes. The identification of extreme resistance in certain isolates, such as *Stenotrophomonas* sp. VITSA1, *Stenotrophomonas pavanii* strain VITSA2, *Acinetobacter* sp. VITSA13, *Klebsiella pneumoniae* strain VITSA14, and *Pandoraea* sp. VITSA19, suggests the presence of novel resistance mechanisms and underscores the need for urgent investigation. These findings emphasize the need for continuous monitoring, improved wastewater treatment strategies, and strict antibiotic stewardship programs to mitigate the risks posed by environmental antibiotic resistance. Future studies should prioritize whole-genome sequencing and functional genomics to uncover resistance mechanisms and evaluate their implications for both public and environmental health.

## Data Availability

The datasets presented in this study can be found in online repositories. The names of the repository/repositories and accession number(s) can be found in the article/Supplementary material.

## Footnotes

^1^https://www.eucast.org/clinical_breakpoints (Accessed February 17, 2025).
