# Supplementary material for: Antibiotic resistance patterns of environmental bacteria from sewage water in Vellore, India: isolation, virulence analysis, and characterization
Source: Front Microbiol. 2025 Aug 12;16:1640369. doi: 10.3389/fmicb.2025.1640369 (PMC12378625; doi:10.3389/fmicb.2025.1640369)
Supplement: Supplementary file 1 [file Data_Sheet_1.pdf]

Additional file:

Antibiotic Resistance Patterns of Environmental Bacteria from Sewage Water in Vellore, India: Isolation, Virulence Analysis and Characterization

Surbhi Kumari Barnwal[1] and Arabi Mohammed Saleh M A[2*]
[1]School of Bio Sciences and Technology, Vellore Institute of Technology, Vellore, Tamil Nadu-632014, India
[2]VIT School of Agricultural Innovations and Advance Learning, Vellore Institute of Technology, Vellore, Tamil Nadu-632014, India

[*]Corresponding author details:
Associate Professor, VIT School of Advance Learning and Innovations, Vellore Institute of Technology, Vellore, Tamil Nadu-632014, India
Email address: amsaleh@vit.ac.in

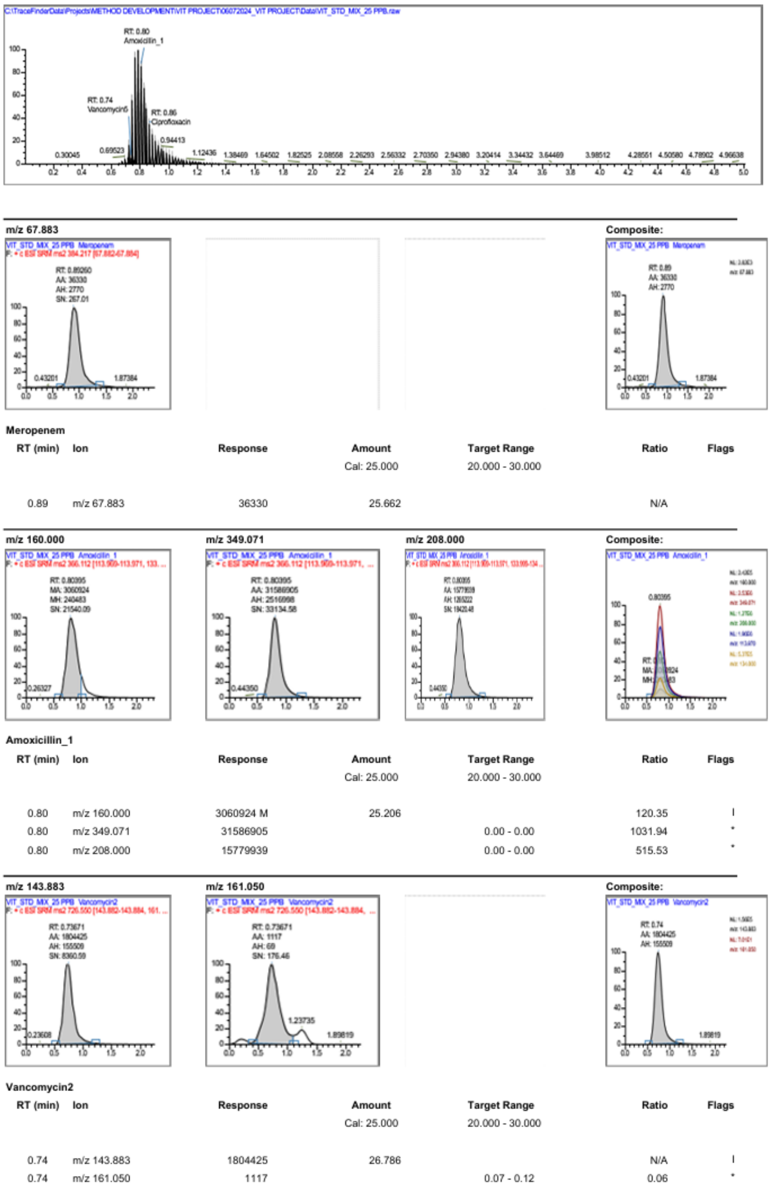

Figure A1. TIC MRM of amoxicillin trihydrate, meropenem trihydrate and vancomycin hydrochloride (25 mg mL^{-1}) analysed using Thermo Scientific TSQ Quantis plus LCMS/MS in ESI+ mode

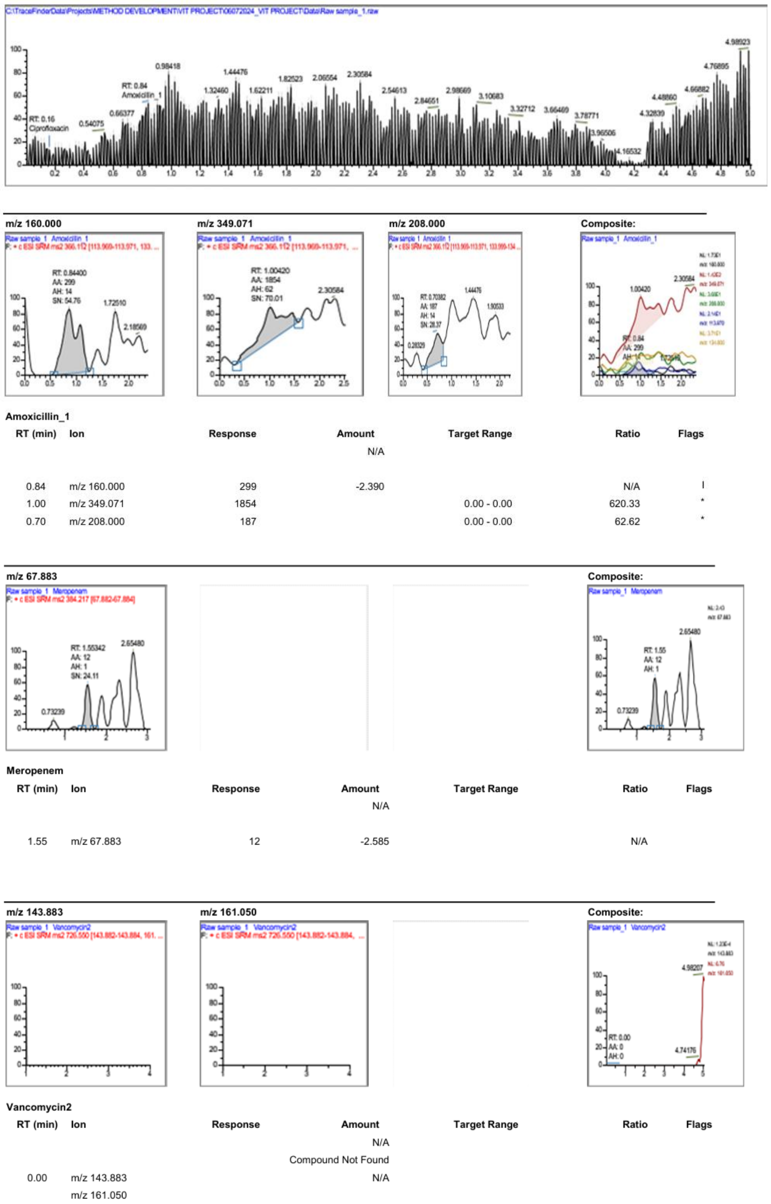

Figure A2. TIC MRM of sample 1 analysed using Thermo Scientific TSQ Quantis plus LCMS/MS in ESI+ mode

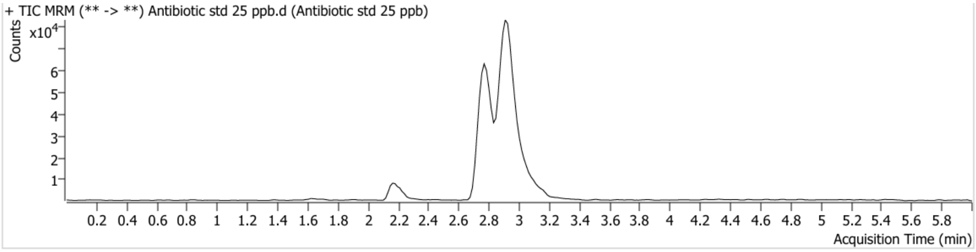

Name	RT	Transition	Resp.	Height	Final Conc. Units
Vancomycin	2.176	726.0 -> 100.1	22115	2655.17	24.5045 ng/ml
Amoxicillin	2.777	366.0 -> 349.1	210418	22083.33	23.3384 ng/ml
Meropenem	2.928	384.0 -> 68.2	353594	36043.63	23.1233 ng/ml

Figure A3. TIC MRM of amoxicillin trihydrate, meropenem trihydrate and vancomycin hydrochloride (25 mg mL^{-1}) analysed using Agilent 6460 Triple Quad 6460, ESI+ mode

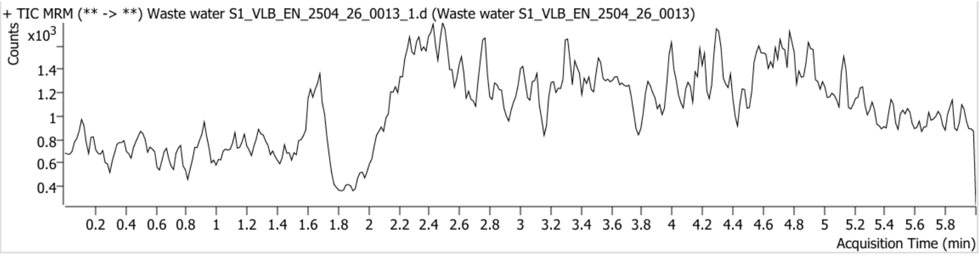

Name	RT	Transition	Resp.	Height	Final Conc. Units
Vancomycin	2.114	726.0 -> 100.1	96	13.66	0.0000 ng/ml
Amoxicillin	2.777	366.0 -> 349.1	1000	183.19	0.3678 ng/ml
Meropenem	3.021	384.0 -> 68.2	314	33.25	0.3726 ng/ml

Figure A4. TIC MRM of sample 2 analysed using Agilent 6460 Triple Quad 6460, ESI+ mode

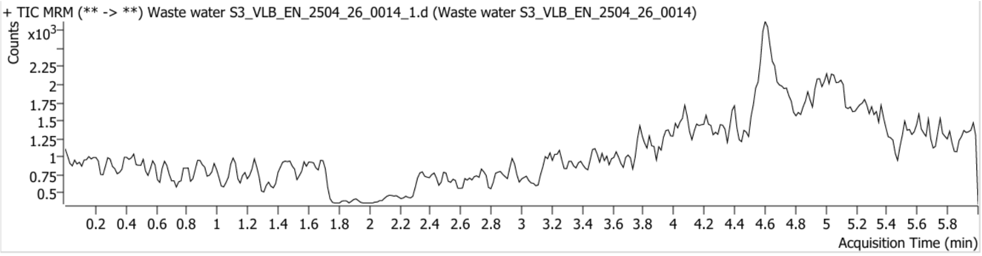

Name	RT	Transition	Resp.	Height	Final Conc. Units
Vancomycin	2.114	726.0 -> 100.1	2	0.00	0.0000 ng/ml
Amoxicillin	2.669	366.0 -> 349.1	211	38.21	0.3516 ng/ml
Meropenem	2.897	384.0 -> 68.2	92	15.55	0.3699 ng/ml

Figure A5. TIC MRM of sample 3 analysed using Agilent 6460 Triple Quad 6460, ESI+ mode

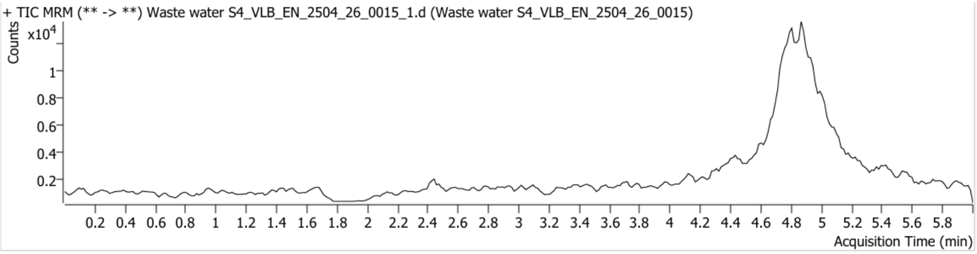

Name	RT	Transition	Resp.	Height	Final Conc. Units
Vancomycin	2.238	726.0 -> 100.1	1	0.11	0.0000 ng/ml
Amoxicillin	2.808	366.0 -> 349.1	170	51.92	0.3507 ng/ml
Meropenem	2.850	384.0 -> 68.2	122	25.45	0.3703 ng/ml

Figure A6. TIC MRM of sample 4 analysed using Agilent 6460 Triple Quad 6460, ESI+ mode

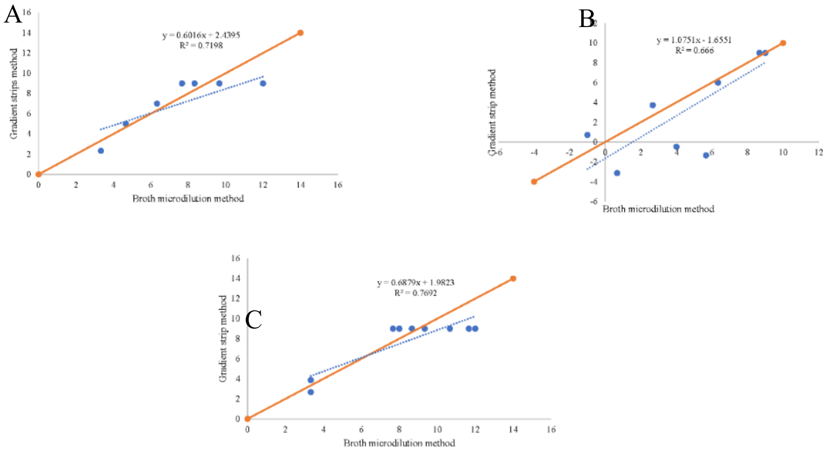

Figure A7. Correlation between mean MIC values (log_2 µg/mL) obtained using broth microdilution and gradient strip methods for amoxicillin. **(A)** Amoxicillin- mean MIC values (log_2 µg/mL) **(B)** Meropenem- mean MIC values (log_2 µg/mL) and **(C)** Vancomycin- mean MIC values (log_2 µg/mL).

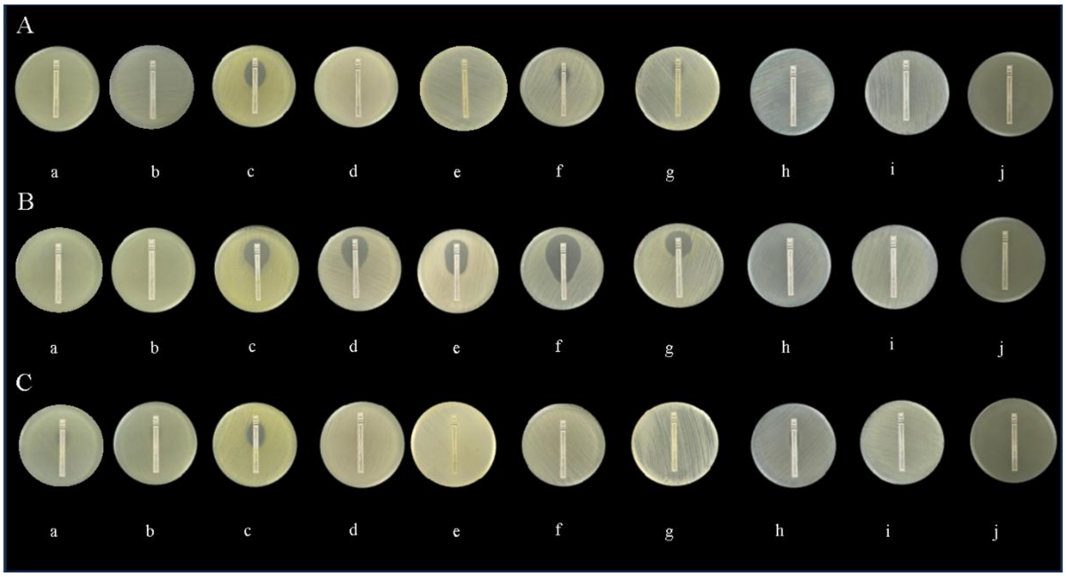

Figure A8. Minimum inhibitory concentration (MIC) gradient strip method results for the bacterial isolates- **(a)** VITSA1, **(b)** VITSA2, **(c)** VITSA3, **(d)** VITSA4, **(e)** VITSA8, **(f)** VITSA11, **(g)** VITSA12, **(h)** VITSA13, **(i)** VITSA14, **(j)** VITSA19 against **(A)** amoxicillin, **(B)** meropenem and **(C)** vancomycin

Table A1. Geographical coordinates of sampling sites.

**Sample**	**Latitude**	**Longitude**
Sample 1	12.93400°N	79.14329°E
Sample 2	12.93359°N	79.14338°E
Sample 3	12.93078°N	79.13647°E
Sample 4	12.93014°N	79.13663°E

Table A2: MIC values (µg/mL) for amoxicillin, meropenem and vancomycin of all bacteria determined via the gradient strip method

Isolate	Amoxicillin MIC	Amoxicillin MIC	Amoxicillin MIC	Meropenem MIC	Meropenem MIC	Meropenem MIC	Vancomycin MIC	Vancomycin MIC	Vancomycin MIC
VITS A1	≥ 512	≥ 512	≥ 512	≥ 512	≥ 512	≥ 512	≥ 512	≥ 512	≥ 512
VITS A2	≥ 512	≥ 512	≥ 512	≥ 512	≥ 512	≥ 512	≥ 512	≥ 512	≥ 512
VITS A3	4	4	8	2	1.5	1.5	4	8	8
VITS A4	≥ 512	≥ 512	≥ 512	1.5	0.5	0.5	≥ 512	≥ 512	≥ 512
VITS A8	≥ 512	≥ 512	≥ 512	0.5	0.25	0.5	≥ 512	≥ 512	≥ 512
VITS A11	32	32	32	0.125	0.094	0.125	≥ 512	≥ 512	≥ 512
VITS A12	128	128	128	16	12	12	≥ 512	≥ 512	≥ 512
VITS A13	≥ 512	≥ 512	≥ 512	≥ 512	≥ 512	≥ 512	16	12	16
VITS A14	≥ 512	≥ 512	≥ 512	64	64	64	≥ 512	≥ 512	≥ 512
VITS A19	≥ 512	≥ 512	≥ 512	≥ 512	≥ 512	≥ 512	≥ 512	≥ 512	≥ 512

Table A3: MIC values (µg/mL) for amoxicillin, meropenem and vancomycin of all bacteria determined via the broth microdilution method

Isolate	Amoxicillin MIC	Amoxicillin MIC	Amoxicillin MIC	Meropenem MIC	Meropenem MIC	Meropenem MIC	Vancomycin MIC	Vancomycin MIC	Vancomycin MIC
VITS A1	≥ 4096	≥ 4096	≥ 4096	≥ 512	≥ 512	≥ 512	256	128	256
VITS A2	≥ 4096	≥ 4096	≥ 4096	≥ 512	≥ 512	≥ 512	256	256	256
VITS A3	8	16	8	0.5	0.5	0.5	8	16	8
VITS A4	512	256	256	64	4	16	≥ 4096	256	≥ 4096
VITS A8	256	256	128	32	128	32	≥ 4096	2048	≥ 4096
VITS A11	64	8	32	32	0.5	0.25	1024	256	256
VITS A12	128	64	64	16	1	16	1024	1024	≥ 4096
VITS A13	2048	1024	256	≥ 512	256	≥ 512	4	16	16
VITS A14	≥ 4096	≥ 4096	≥ 4096	64	64	128	512	512	1024
VITS A19	≥ 4096	≥ 4096	≥ 4096	≥ 512	≥ 512	≥ 512	≥ 4096	≥ 4096	≥ 4096

Table A4: MIC log_2 values, mean, SD, SE (µg/mL) for amoxicillin, meropenem and vancomycin of all bacteria determined via the gradient strip method (two-factor ANOVA with triplicates for all antibiotics was performed, significant at $p < 0.05$)

Isolate	Amoxicillin	Amoxicillin	Amoxicillin	Mean	SD	SE	Meropenem	Meropenem	Meropenem	Mean	SD	SE	Vancomycin	Vancomycin	Vancomycin	Mean	SD	SE
VIT SA1	9	9	9	9	0	0	9	9	9	9	0	0	9	9	9	9	0	0
VIT SA2	9	9	9	9	0	0	9	9	9	9	0	0	9	9	9	9	0	0
VIT SA3	2	2	3	2.33	0.58	0.33	1	0.58	0.58	0.72	0.24	0.14	2	3	3	2.67	0.58	0.33
VIT SA4	9	9	9	9	0	0	0.58	-1	-1	-0.47	0.91	0.53	9	9	9	9	0	0
VIT SA8	9	9	9	9	0	0	-1	-2	-1	-1.33	0.58	0.33	9	9	9	9	0	0
VIT SA11	5	5	5	5	0	0	-3	-3.41	-3	-3.14	0.24	0.14	9	9	9	9	0	0
VIT SA12	7	7	7	7	0	0	4	3.58	3.58	3.72	0.24	0.14	9	9	9	9	0	0
VIT SA13	9	9	9	9	0	0	9	9	9	9	0	0	4	3.58	4	3.86	0.24	0.14
VIT SA14	9	9	9	9	0	0	6	6	6	6	0	0	9	9	9	9	0	0
VIT SA19	9	9	9	9	0	0	9	9	9	9	0	0	9	9	9	9	0	0

Table A5: MIC log_2 values, mean, SD, SE (µg/mL) for amoxicillin, meropenem and vancomycin of all bacteria determined via the broth microdilution method (two-factor ANOVA with triplicates for all antibiotics was performed, significant at $p < 0.05$)

Isolate	Amoxicillin	Amoxicillin	Amoxicillin	Mean	SD	SE	Meropenem	Meropenem	Meropenem	Mean	SD	SE	Vancomycin	Vancomycin	Vancomycin	mean	SD	SE
VITSA1	12	12	12	12	0	0	9	9	9	9	0	0	8	7	8	7.67	0.57	0.33
VITSA2	12	12	12	12	0	0	9	9	9	9	0	0	8	8	8	8	0	0
VITSA3	3	4	3	3.33	0.58	0.33	-1	-1	-1	-1	0	0	3	4	3	3.33	0.58	0.33
VITSA4	9	8	8	8.33	0.58	0.33	6	2	4	4	2	1.15	12	8	12	10.67	2.31	1.33
VITSA8	8	8	7	7.67	0.58	0.33	5	7	5	5.67	1.15	0.67	12	11	12	11.67	0.58	0.33
VITSA11	6	3	5	4.67	1.53	0.88	5	-1	-2	0.67	3.79	2.19	10	8	8	8.67	1.15	0.67
VITSA12	7	6	6	6.33	0.58	0.33	4	0	4	2.67	2.31	1.33	10	10	12	10.67	1.15	0.67
VITSA13	11	10	8	9.67	1.53	0.88	9	8	9	8.67	0.58	0.33	2	4	4	3.33	1.15	0.67
VITSA14	12	12	12	12	0	0	6	6	7	6.33	0.58	0.33	9	9	10	9.33	0.58	0.33
VITSA19	12	12	12	12	0	0	9	9	9	9	0	0	12	12	12	12	0	0

Table A6. Paired t-test

Antibiotics	Amoxicillin	Meropenem	Vancomycin
p-value	0.09	0.20	0.19
